# DIGITAL LITERACY CHANGES OVER TIME: IMPLICATIONS FOR LIFE SATISFACTION IN OLDER ADULTS

**DOI:** 10.1093/geroni/igae098.2558

**Published:** 2024-12-31

**Authors:** Miseon Kang, Seolah Lee, Inhye Jung, Hyo Jung Lee

**Affiliations:** Yonsei University, Seoul, Republic of Korea; Yonsei University, Seoul, Republic of Korea; Yonsei University, Seoul, Republic of Korea; Yonsei University, Seoul, Republic of Korea

## Abstract

Rapid digitalization of society requires people to utilize digital technology in daily lives, and the COVID-19 pandemic accelerated this situation. Although older adults are known to be unfamiliar with using digital technology, their digital literacy can vary according to their life experiences. This study aimed to identify patterns of digital literacy changes in older adults before and after the COVID-19 pandemic and examined how life satisfaction differs based on subtypes of digital literacy changes over time. Data were drawn from the ‘Media Panel’ by the Korea Information Society Development Institute. The final sample was 1,924 individuals over 65 who responded from 2019 to 2021 and maintained their living arrangements (Living alone, Couples only, Couples with children, and Couples with their parents and children). Latent Class Growth Analysis and multiple regression analyses were employed. The patterns of older adults’ digital literacy changes were classified into four subtypes: ‘Low-level increase group (60.9%),’ ‘Medium-level increase group (29.8%),’ ‘High-level maintenance group (5.0%),’ and ‘Rapid increase group (4.2%). The other three groups had higher life satisfaction than the low-level increase group. Findings imply that the majority of older adults saw a rise in their digital literacy skills during the COVID-19 pandemic. The heterogeneity in digital literacy levels among older adults offers valuable insight into the need for interventions that cater to different digital skill levels, potentially impacting their quality of life. Particularly, in order to enhance their overall well-being, it is important to better understand the key features of individuals in each identified group.

